# T227. THE METABOTROPIC GLUTAMATE RECEPTOR SUBTYPE 1 REGULATES STRIATAL DOPAMINE RELEASE VIA AN ENDOCANNABINOID-DEPENDENT MECHANISM: IMPLICATIONS FOR THE TREATMENT OF SCHIZOPHRENIA

**DOI:** 10.1093/schbul/sby016.503

**Published:** 2018-04-01

**Authors:** Samantha Yohn, Daniel Covey, Daniel Foster, Mark Moehle, Jordan Galbraith, Joseph Cheer, Craig Lindsley, P Jeffrey Conn

**Affiliations:** 1 Vanderbilt University; 2 University of Maryland School of Medicine; 3 Vanderbilt University Medical Center

## Abstract

**Background:**

Clinical and preclinical studies suggest that selective activators of the muscarinic M4 receptor have exciting potential as a novel approach for treatment of schizophrenia. M4 reduces striatal dopamine (DA) though release of endocannabinoids (eCB), providing a mechanism for local effects on DA signaling in the striatum. M4 signals through Gαi/o and does not couple to Gαq/11 or induce calcium (Ca++) mobilization. This raises the possibility that M4-induced eCB release and inhibition of DA release may require co-activation of another receptor that activates Gαq/11. If so, this receptor could provide a novel target that may be more proximal to inhibition of DA release. Interestingly, the group 1 metabotropic glutamate (mGlu) receptors (mGlu1 and Glu5), couple to Gαq/11 and activate eCB signaling in multiple brain regions.

**Methods:**

We tested the hypothesis that M4-induced reductions in DA release and subsequent antipsychotic-effect requires co-activation of group 1 mGlu receptors. The effect of M4 activation on electrically-evoked DA release in striatal slices was assessed using fast-scan cyclic voltammetry (FSCV) in the absence or presence of selective negative allosteric modulators (NAMs) of group 1 mGlu receptor subtypes. To evaluate the potential role of mGlu1, we determined the effects of a selective mGlu1 positive allosteric modulators (PAMs) on striatal DA release and antipsychotic-like activity in rodent models that are dependent on increased DA transmission. Since reductions in DA signaling, including D1 signaling have been implicated in reduced motivation, we also determined the effects of an mGlu1 PAM, M4 PAM, and the typical antipsychotic haloperidol on motivational responding in a progressive ratio (PR) schedule.

**Results:**

We now present exciting new data in which we found that activation of mGlu1 through application of exogenous agonists or selective stimulation of thalamostriatal afferents induces a reduction of striatal DA release and that selective mGlu1 PAMs have robust antipsychotic-like effects in rodent models. Interestingly, our studies also suggest that mGlu1 activation is required for M4 PAM-induced inhibition of DA release and antipsychotic-like effects. However, in contrast to available antipsychotic agents, the present results and previous studies suggest that mGlu1 and M4 PAMs reduce DA signaling through local release of an eCB from striatal SPNs and activation of CB2 receptors on neighboring DA terminals to reduce DA release. While these studies suggest that the effects of M4 PAMs on DA release require activation of mGlu1, we have also found that these targets have important differences. Most notably, M4 PAMs also directly inhibits D1 signaling in D1-SPN terminals in the substatnia nigra pars reticulata (SNr). Unlike M4, mGlu1 does not directly inhibit DA D1 receptor signaling and does not induce behavioral changes that could be associated with negative symptoms.

**Discussion:**

Our findings are especially interesting in light of recent findings that multiple loss of function single nucleotide polymorphisms (SNPs) in the human gene encoding mGlu1 (GRM1) are associated with schizophrenia, and points to GRM1/mGlu1 as a gene within the “druggable genome” that could be targeted for treatment of schizophrenia. Recent clinical imaging studies suggesting that symptoms in schizophrenia patients are associated with selective increases in striatal DA signaling and while extrastriatal regions display hypo-dopaminergic function; thus, mGlu1 and M4 PAMs may provide a mechanism for selective inhibition of DA release in striatal regions that are important for antipsychotic efficacy, without further disruptions in extrastriatal DA signaling.

